# Nociceptive DRG neurons express muscle lim protein upon axonal injury

**DOI:** 10.1038/s41598-017-00590-1

**Published:** 2017-04-04

**Authors:** Evgeny Levin, Anastasia Andreadaki, Philipp Gobrecht, Frank Bosse, Dietmar Fischer

**Affiliations:** 1 Division of Experimental Neurology, Department of Neurology, Heinrich-Heine-University of Düsseldorf, Merowingerplatz 1a, 40225 Düsseldorf, Germany; 2 Molecular Neurobiology Laboratory, Department of Neurology, Heinrich-Heine-University Moorenstrasse 5, 40225 Düsseldorf, Germany

## Abstract

Muscle lim protein (MLP) has long been regarded as a cytosolic and nuclear muscular protein. Here, we show that MLP is also expressed in a subpopulation of adult rat dorsal root ganglia (DRG) neurons in response to axonal injury, while the protein was not detectable in naïve cells. Detailed immunohistochemical analysis of L4/L5 DRG revealed ~3% of MLP-positive neurons 2 days after complete sciatic nerve crush and maximum ~10% after 4–14 days. Similarly, in mixed cultures from cervical, thoracic, lumbar and sacral DRG ~6% of neurons were MLP-positive after 2 days and maximal 17% after 3 days. In both, histological sections and cell cultures, the protein was detected in the cytosol and axons of small diameter cells, while the nucleus remained devoid. Moreover, the vast majority could not be assigned to any of the well characterized canonical DRG subpopulations at 7 days after nerve injury. However, further analysis in cell culture revealed that the largest population of MLP expressing cells originated from non-peptidergic IB4-positive nociceptive neurons, which lose their ability to bind the lectin upon axotomy. Thus, MLP is mostly expressed in a subset of axotomized nociceptive neurons and can be used as a novel marker for this population of cells.

## Introduction

Muscle lim protein (MLP), also known as cysteine rich protein 3 (CRP3, CSRP3), is a member of the family of cysteine rich proteins (CRP). Since its discovery, MLP was supposedly expressed exclusively in muscle tissue. Although rat dorsal root ganglion (DRG) neurons reportedly express Mlp-RNA, the protein was never detected in these cells so that its translation remained questionable^1, 2^. However, we recently found MLP transiently expressed in embryonic and postnatal, but not in mature retinal amacrine cells^3^, suggesting that this protein may be involved in neuronal processes.

Previously, MLP protein is located in the cytosol and/or nucleus of cardiac and skeletal myocytes. There, it is essential for diverse biological processes, such as myocyte differentiation^4, 5^, response to mechanical stress^6, 7^, modulation of the actin-cytoskeleton^8^, maintenance of the cytoarchitecture^9^ and metabolism^10^. This multifunctional role was attributed to MLP’s ability to associate with numerous intracellular interaction partners via its two LIM domains^11–13^. Nuclear MLP accumulation was primarily associated with differentiation or mechanical stress sensing in muscle cells^4, 13, 14^.

DRG are clusters of highly heterogeneous populations of primary sensory neurons^15–17^. These neurons differ substantially in size, gene expression, electrophysiological properties^18–21^ and the type of sensory information they convey. Small diameter neurons with unmyelinated C-fibers are predominantly nociceptors involved in the transduction of pain stimuli^22^ that can be further subdivided into peptidergic and non-peptidergic neurons. While peptidergic nociceptors express neuropeptides such as calcitonin gene-related peptide (CGRP) or substance P (SP)^23, 24^, non-peptidergic neurons can instead be labelled by Isolectin B4 (IB4) from *Griffonia simplicifolia* ^25^. In comparison, large diameter neurons, which are involved in proprioception and mechanosensation, bear heavily myelinated A-fibers and express the heavy chain of neurofilament (200 kDa NF)^20, 26, 27^. Although CGRP/SP, IB4 and 200 kDa NF are commonly used to distinguish DRG subpopulations, the presence of these markers might also overlap in some neuronal populations. For instance, ~30% of IB4-positive DRG neurons in rats are reportedly immunoreactive for CGRP while ~45% of CGRP-positive neurons also bind IB4^28^.

Despite the detection of Mlp-mRNA^2^, expression of the protein has so far been elusive in adult neurons. The current study provides first evidence that MLP protein is actually induced in some adult neurons upon axotomy. More specifically, MLP was mainly detected in non-peptidergic, nociceptive DRG neurons that concurrently and inversely lost their ability to bind the IB4 upon axonal injury. Thus, MLP can be used as a novel marker for this subpopulation of neurons.

## Materials and Methods

### Animals

All experimental procedures were approved by the local animal care committee in Recklinghausen (LANUV, Germany) and conducted in compliance with federal and state guidelines for animal experiments in Germany (approval number: 84-02.04.2015.A290). Rats were maintained on a 12-hour light/dark cycle with *ad libitum* access to food and water. In total 24 rats were used. Animals were killed either by inhalation of CO_2_ for preparations of cell cultures, Western blot lysates and mRNA isolations or by intraperitoneal application of ketamine (60–80 mg/kg; Pfizer) and xylazine (10–15 mg/kg; Bayer) and perfusion through the heart with cold PBS (Gibco) followed by paraformaldehyde (Sigma) (4% PFA in PBS) for immunohistochemical analyses.

### Sciatic nerve crush

In total 18 adult Wistar male rats (200–230 g) were anesthetized by intraperitoneal injections of ketamine (60–80 mg/kg) and xylazine (10–15 mg/kg). A skin incision of about 10 mm was made over the left gluteal region. The ischiocrural musculature was carefully spread with minimal tissue damage to expose the sciatic nerve from the sciatic notch to the point of trifurcation. Severe axonotmetic crush injury was performed proximal to the tibial and peroneal divisions for 30 s using Dumont #5 forceps (Hermle). No crush injury was performed in sham-operated animals. For fluorogold tracing of neurons, the sciatic nerve was re-exposed 5 days after the initial surgery and cut proximal to the crush site. A piece of gel foam soaked in aqueous fluorogold solution (Thermo Fischer) was placed at the proximal stump prior to suturing the skin. Animals were sacrificed 2 days thereafter.

### RNA isolation and quantitative real-time PCR

Total RNA was isolated from L4 and L5 DRG using the RNeasy kit (Qiagen) according to the manufacturer’s protocol and 40 ng RNA were reverse transcribed using the Superscript II kit (Invitrogen). Quantification of Mlp expression was performed relative to the endogenous housekeeping gene glyceraldehyde 3-phosphate dehydrogenase (Gapdh) using SYBR Green PCR Master Mix (Applied Biosystems) and QuantiTect primers Rn_Csrp3_1_SG (order-id: QT00183708) and Rn_Gapdh_1_SG (order-id: QT00199633; QuantiTect Primer Assay; Qiagen) and the Applied Biosystems 7500 real-time PCR System. DRG-derived cDNA was amplified during 45 cycles with amplification efficiency of about 100% according to the manufacturer’s protocol. All reactions were performed in duplicate and at least three independent samples (3 animals) were analyzed per experimental group. Same numbers of sham-operated animals were used as uninjured controls. Relative Mlp expression was calculated using the comparative threshold cycle method (ΔΔC_t_)^29^. Specificity of PCR products was determined and verified for each run using the dissociation curve analysis feature of the Applied Biosystems 7500 software. Data are presented as means ± SEM. Data were tested for normal distribution and significances of intergroup differences calculated with GraphPad Prism software using Student’s t-test.

### MLP knockdown in HEK293 cells

Rat MLP cDNA was cloned into pcDNA3.1/V5-His Topo vector (Thermo Fischer). AVV-U6-EGFP plasmid containing scrambled shRNA was obtained from Vector Biolabs. Mlp-specific shRNA (5′-GATCCGGTTTACCATGCAGAAGAAATCTCGAG-ATTTCTTCTGCATGGTAAACCTTTT-AGATCTA-3′) was also cloned into AVV-U6-EGFP. HEK293 cells were seeded at ~3–5 × 10^4^ cells per well in 96-well plates and co-transfected with MLP cDNA and shRNA-constructs using Lipofectamin 2000 (Thermo Fischer) the next day. Cells were stained for MLP protein using a goat anti-MLP antibody (1:500; C9001-23; US Biological; RRID: AB_2087783) after further 24 hours of incubation.

### Immunohistochemistry and image analysis

In total, 8 rats were anesthetized and perfused through the heart with cold PBS followed by paraformaldehyde (4% PFA in PBS). L4/L5 DRG and sciatic nerves were isolated, post-fixed for 3 hours in 4% PFA, transferred to 30% sucrose overnight (4 °C) and embedded in KP cryo-compound (Klinipath). Serial sagittal sections (thickness of 14 µm) through the whole DRG including parts of the peripheral and central roots were prepared using a cryostat (Leica), thaw-mounted onto Superfrost plus glass slides (VWR) and stored at −20 °C until further use. For immunostaining, sections were exposed to 100% methanol for 10 min and then blocked with 2% bovine serum albumin (BSA, Sigma) and 5% donkey serum (Sigma) in PBS containing 0.05% Tween 20 (Sigma). Sections were incubated with primary antibodies dissolved in blocking solution for 16 hours. Primary antibodies included goat anti-MLP (1:400; C9001-23; US Biological; RRID: AB_2087783), mouse anti-MLP^30^ [a kind gift from Dr. Geier, Max Delbrück Center for Molecular Medicine, Berlin, Germany], mouse anti-βIII-tubulin (1:1000; clone TUJ1; MMS-435P; Biolegend; RRID: AB_2313773), rabbit anti-βIII-tubulin (1:1000; clone TUJ1; MRB-435P; Biolegend; RRID: AB_10175616), rabbit anti-CGRP (1:100; BML-CA1134-0025; Enzo; RRID: AB_2050884), rabbit anti-Substance P (1:500; ABIN617879; ImmunoStar; RRID: AB_572266), mouse anti-200 kDa neurofilament (1:1000; ab78158, Abcam; RRID: AB_1566479), mouse anti-ATF3 (1:200; ab58668; Abcam; RRID: AB_879578) and rabbit anti-tyrosine hydroxylase (1:500; NB300-109; Novus Biologicals; RRID: AB_10077691). Sections were washed three times with PBS for 10 min, incubated with secondary antibodies dissolved in blocking solution as described above for 1 h and then washed three times with PBS. Secondary antibodies included donkey anti-mouse, donkey anti-rabbit and donkey anti-goat antibodies conjugated to either Alexa Fluor 488, Alexa Fluor 594 or Alexa Fluor 405 (1:1000; Thermo Fisher). For IB4 staining, some sections were subsequently incubated with IB4 lectin (FITC-conjugated, 25 µg/ml in PBS, L2895, Sigma) for 2 h. Stained sections were embedded in Mowiol mounting medium and the whole DRG section containing 300–600 neurons was photographed using a fluorescent microscope (Observer.D1, Zeiss) at 200x magnification (6–10 images per DRG section). For size determination of MLP-positive neurons, the cross-sectional cell area was measured at the level of the nucleus using ImageJ Software. Values were grouped into 50 µm^2^ bins and respective proportions of MLP-positive neurons in each bin depicted as histogram. For the quantification of neuronal subpopulations, CTCF (corrected total cell fluorescence) values were calculated for strongly subtype marker-positive neurons with visible nuclei co-stained with βIII-tubulin using the ImageJ software and the following formula: CTCF = integrated density of stained cell − (mean background fluorescence × cell area). Only labeled cells with CTCF of ≥100000 were counted. Four randomly selected sections per DRG were analyzed for each neuronal subpopulation. MLP fluorescence intensities in nuclei and cytoplasm were quantified using confocal images obtained with the Leica TCS SP8 confocal laser scanning microscope at 200x magnification. Integrated densities of same sized areas were measured in nuclei and cytoplasm and corrected for background fluorescence. Data were tested for normal distribution and significances of intergroup differences calculated with GraphPad Prism software using either one-way analysis of variance (ANOVA) with Holm-Sidak *post hoc* test or Student’s t-test and are presented as means ± SEM.

### DRG neuron cultures

DRG neurons were isolated from 6 adult male rats as previously described^31–33^. In brief, DRG were harvested, incubated in Dulbecco’s modified Eagle medium (DMEM) containing 0.25% trypsin/EDTA (Thermo Fisher) and 0.3% collagenase type IA (Sigma) and mechanically dissociated. Cells were re-suspended in DMEM supplemented with 10% fetal bovine serum (FBS, GE Healthcare) and 500 U/ml penicillin/streptomycin (BioChrom) and plated into 96-well plates coated with poly-D-lysine (0.1 mg/ml, molecular weight <300,000 Da, Sigma) and 20 μg/ml laminin (Sigma). Some cultures were treated with 50 ng/ml human recombinant glial-derived neurotrophic factor (GDNF, Peprotech) immediately before plating and on day 3 in culture. Cells were cultured at 37 °C in 5% CO_2_ for up to 5 days, fixed in 4% PFA for 25 min and then permeabilized in 100% methanol for 10 min. Cultures were then blocked with 2% bovine serum albumin (BSA, Sigma) and 5% donkey serum (Sigma) in PBS containing 0.05% Tween 20 (Sigma) and incubated with primary antibodies dissolved in blocking solution for 16 hours. Primary antibodies included goat anti-MLP (1:400; C9001-23; US Biological; RRID: AB_2087783), mouse anti-βIII-tubulin (1:1000; clone TUJ1; MMS-435P; Biolegend; RRID: AB_2313773), rabbit anti-βIII-tubulin (1:1000; clone TUJ1; MRB-435P; Biolegend; RRID: AB_10175616), rabbit anti-CGRP (1:100; BML-CA1134-0025; Enzo; RRID: AB_2050884), mouse anti-200 kDa neurofilament (1:1000; ab78158, Abcam; RRID: AB_1566479). Plates were washed three times with PBS for 10 min, incubated with secondary antibodies dissolved in the blocking solution as described above for 1 h and then washed three times with PBS. Secondary antibodies included donkey anti-mouse, donkey anti-rabbit and donkey anti-goat antibodies conjugated to either Alexa Fluor 488, Alexa Fluor 594 or Alexa Fluor 405 (1:1000; Thermo Fisher). For IB4 staining some sections were subsequently incubated with IB4 lectin (FITC-conjugated, 25 µg/ml in PBS, L2895, Sigma) for 2 h. Percentages of DRG neurons stained by the respective marker(s) were quantified using a fluorescent microscope (Observer.D1, Zeiss) in two independent experiments with at least three replicate wells for each subpopulation. Data were tested for normal distribution and significances of intergroup differences calculated with GraphPad Prism software using either one-way analysis of variance (ANOVA) with Holm-Sidak *post hoc* test or Student’s t-test and are presented as means ± SEM.

### Western blot assay

For protein lysate preparation, 2 rats with sciatic nerve crush (SNC) and 2 rats with sham-surgery were killed and DRG isolated. DRG were homogenized in lysis buffer (20 mM Tris/HCl pH 7.5, 10 mM KCl, 250 mM sucrose, 10 mM NaF, 1 mM DTT, 0.1 mM Na3VO4, 1% Triton X-100, 0.1% SDS) with protease inhibitors (Calbiochem, Darmstadt, Germany) and phosphatase inhibitors (Roche, Basel, Switzerland) using 5 sonication pulses at 40% power (Bandelin Sonoplus). Lysates were cleared by centrifugation in an Eppendorf tabletop centrifuge at 5000 rpm for 10 min at 4 °C. Lysates of heart were used as a positive control. Moreover, cell-lysates from HEK293 cells transfected with MLP expression plasmid and either scrambled control shRNA or Mlp-shRNA were prepared to verify the specificity of the antibody. Proteins were separated by sodium-dodecyl-sulfate-polyacrylamide gel electrophoresis (SDS-PAGE), using Mini TGX gels (10%, BioRad, Hercules, USA) according to standard protocols and transferred to polyvinylidene fluoride (PVDF) membranes (Bio-Rad). Blots were blocked in 5% dried milk with 1% bovine serum albumin (BSA) in phosphate- buffered saline with 0.05% Tween 20 (PBS-T) (Sigma) and incubated with antibodies against mouse anti-β-actin (1:4000; A5441; Sigma; RRID: AB_476744), or goat anti-MLP (1:500; C9001-23; US Biological; RRID: AB_2087783) at 4 °C overnight. All antibodies were diluted in 5% dried milk in PBS-T. Bound anti-MLP-antibody was visualized with anti-goat immunoglobulin G (IgG) secondary antibody conjugated to horseradish peroxidase (Sigma, 1:30 000) and the antigen-antibody complexes were detected by enhanced chemiluminescence (Biozyme) on a FluorChem E detection system (ProteinSimple). Bound anti-actin-antibody was visualized with anti-mouse infrared dye secondary antibody (IRDey 800 CW; LI-COR; 1:20 000) using Odyssey Infrared Imaging System (LI-COR).

## Results

### Induction of MLP expression in a subset of DRG neurons upon axotomy

**Figure 1:**
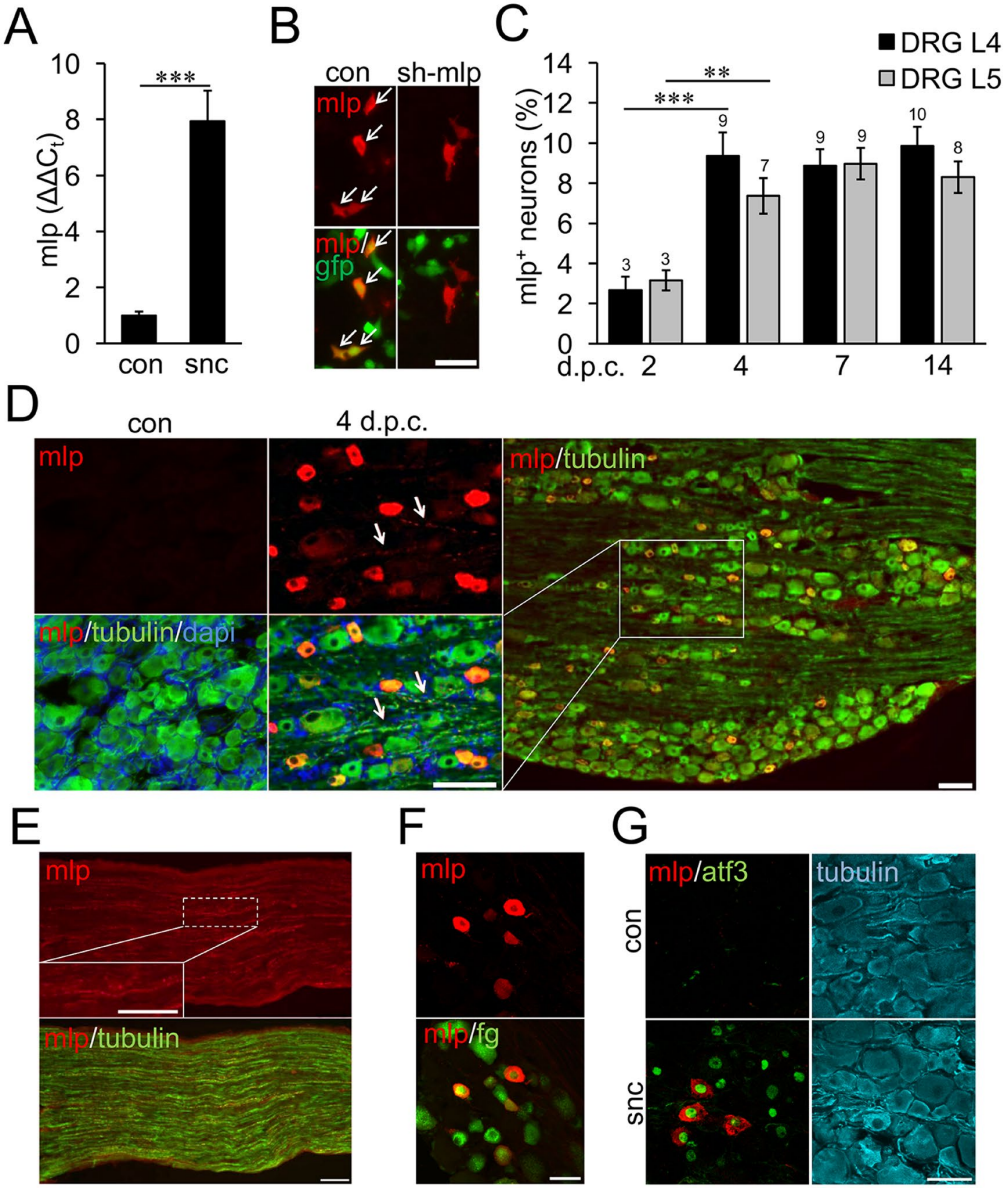
MLP is expressed in a subpopulation of DRG neurons. (**A**) Quantitative RT-PCR reveals strong induction of Mlp expression in adult male rat L4/L5-DRG 7 days after sciatic nerve crush (snc) in comparison to sham-operated controls (con). Samples of 3 animals per group (snc and con) were used. The Ct values for Mlp were in the range of 24 to 30 cycles and the Ct values for Gapdh of 18–19 cycles. Treatment effect: ***p < 0.001. (**B**) HEK293 cells were co-transfected with MLP expression plasmid and either scrambled control shRNA (con) or Mlp-shRNA (sh-Mlp). GFP expression indicates shRNA-transfected cells and MLP was detected 24 hours after transfection. Mlp-shRNA knocked down MLP expression. Scale bar: 50 µm. (**C**) Quantification of MLP-positive (mlp^+^) L4 and L5 DRG neurons at 2, 4, 7 and 14 days post sciatic nerve crush (d.p.c.). Data represent mean percentages ± SEM of all βIII-tubulin-stained neurons on DRG sections as in (**D**). Two male rats and 4 sections per DRG were used for each group. Treatment effects: **p < 0.01, ***p < 0.001. (**D**) Immunohistochemical analysis of MLP expression on DRG sections of sham-operated controls (con) and 4 d.p.c. The sections were stained for MLP (red), βIII-tubulin (green) and DAPI (blue). MLP was detected in a subpopulation of βIII-tubulin-positive neurons and their axons (arrows). Scale bars: 50 µm. (**E**) MLP expression (red) was also detected in βIII-tubulin-positive axons (green) on proximal sciatic nerve sections at 4 d.p.c. The insert shows a higher magnification of a MLP-positive axon. Scale bars: 100 µm. (**F**) MLP immunostaining on DRG sections 7 days after sciatic nerve cut and retrograde fluorogold (fg) labeling. MLP expression was only detected in fg-positive injured neurons. Scale bar: 50 µm. (**G**) DRG sections of sham-operated controls (con) and 4 d.p.c were stained for MLP (red), ATF3 (green) and βIII-tubulin (cyan). ATF3 expression indicated axotomized DRG neurons. MLP was only detected in ATF3-positive neurons. Scale bar: 50 µm.

**Figure 2:**
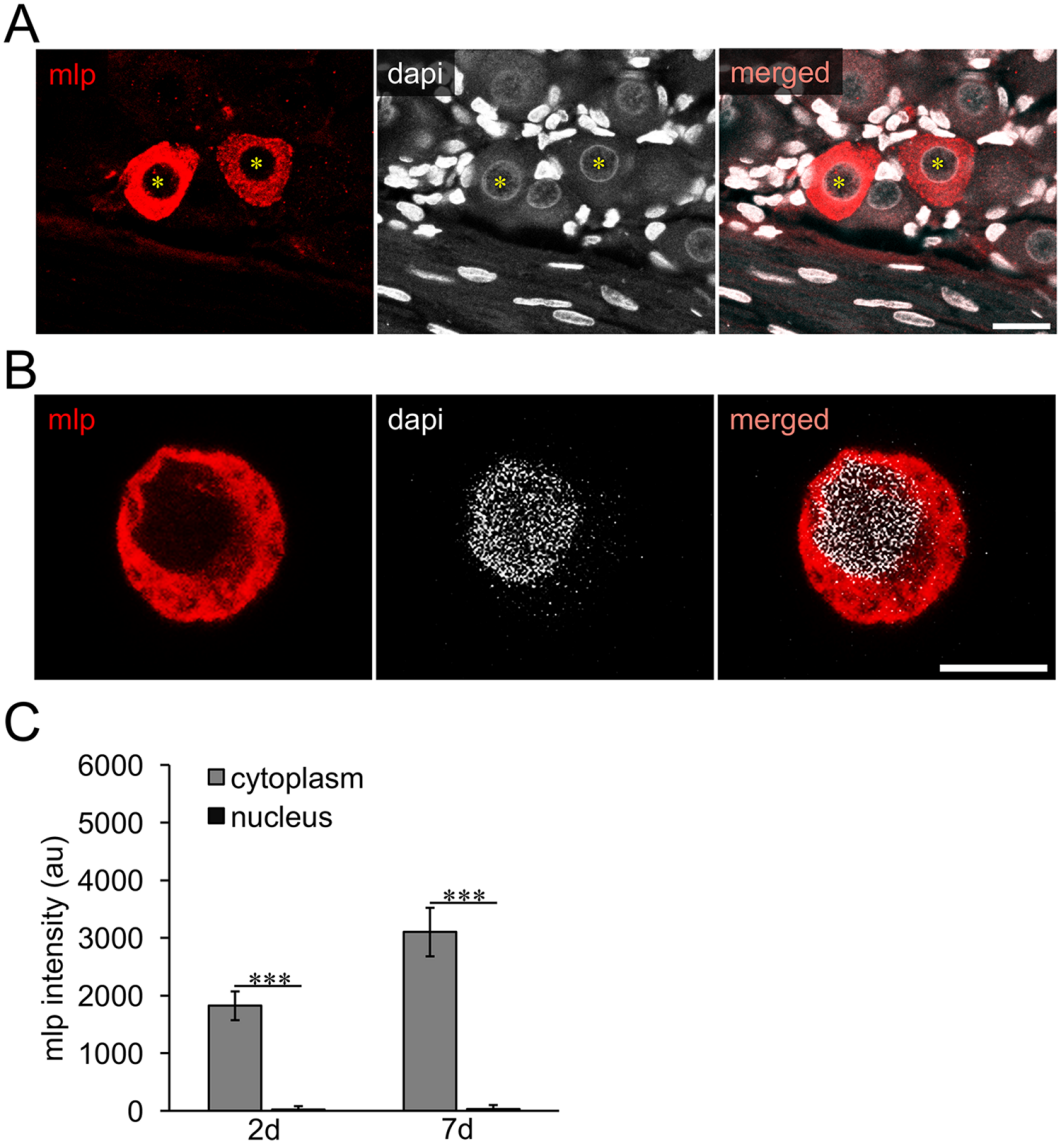
MLP is located in the cytosol, but not the nucleus. (**A**) Confocal images (4 merged z-stacks of 1.04 µm each) of MLP (red) and DAPI (white) stained DRG section 7 days after sciatic nerve crush. MLP was detected in the cytoplasm, but not in DAPI-positive nuclei of DRG neurons (stars). Scale bar: 20 µM. (**B**) Confocal image (3 merged z-stacks of 0.956 µm) of a MLP (red) and DAPI (white) stained DRG neuron after 3 days in culture. MLP was not detected in nuclei of cultured DRG neurons. Scale bar: 10 µM. (**C**) Intensities of MLP signals were quantified on confocal images 2 and 7 days after sciatic nerve crush stained as described in (**A**) in nuclei and cytoplasm of MLP-positive neurons. Data are indicated in arbitrary units (au) for background-corrected fluorescence intensities. Effects: ***p < 0.001.

Although Mlp mRNA was reportedly detected in axotomized dorsal root ganglia (DRG) of adult rats^1, 2^, it remained unknown whether these neuronal Mlp transcripts are actually translated into protein. In order to confirm this previous finding, we first performed quantitative Mlp RT-PCR on mRNA isolated from adult rat L4 and L5 DRG as these project axons into the sciatic nerve. Indeed, Mlp expression was strongly induced (~8-fold increase) at 7 days after sciatic nerve crush (SNC) in comparison to uninjured nerves (Fig. 1A). We then tested the applicability and specificity of a commercially available polyclonal MLP antibody (C9001-23 from US Biological) for immunohistochemistry and Western-Blot analysis. To this end, HEK293 cells were co-transfected with a MLP expression plasmid and either Mlp-shRNA or scrambled control shRNA. Immunocytochemistry of these cultures revealed strong and clear MLP staining in control-shRNA transfected cells, but no signal was detectable upon Mlp-shRNA expression (Fig. 1B). Similarly, the antibody detected a strong band at the size of 21 kD in heart-lysate and MLP-expressing HEK-lysates, which was markedly reduced by shRNA (Suppl. Fig. 1A). Therefore, this polyclonal serum and a previously characterized proprietary monoclonal antibody^30^ were deemed suitable for immunohistochemical studies as well as Western-Blot analysis and subsequently used to specifically stain MLP on DRG sections. No staining was observed in control L4/L5 DRG without prior SNC, but a pronounced signal was detected in some of the βIII-tubulin-positive neurons 4 days post crush (d.p.c.) (Fig. 1C,D). These results were verified by Western-Blot analysis (Suppl. Fig. 1A). MLP staining was found in cell somata as well as in axons and some axonal profiles were accordingly stained in longitudinal sciatic nerve sections (Fig. 1E). The time course of MLP induction was established by the quantification of MLP-positive neurons in L4 and L5 DRG at different time points after sciatic nerve crush. Only 3% of all βIII-tubulin-positive neurons were also stained for MLP at 2 d.p.c. (initial stage of regeneration) and the level of 9% reached at 4 d.p.c. did not further increase up to 14 d.p.c. (later stage) (Fig. 1C). Moreover, retrograde fluorogold labeling from the cut sciatic nerve (Fig. 1F) and co-staining of MLP-positive neurons with neuronal injury marker ATF3 (Fig. 1G) confirmed that MLP was only expressed in axotomized DRG neurons (in ~15% of ATF-3 positive cells). Thus, MLP expression was induced in a relatively small number of axotomized DRG neurons. In addition, detailed confocal microscopy revealed that neuronal MLP expression was restricted to the cytoplasm and, in contrast to myocytes, not found in nuclei. Quantification of MLP-expression on histological DRG sections (Fig. 2A,C) as well as staining of isolated DRG neurons in culture (Fig. 2B) featured nuclei devoid of MLP protein. Therefore, induction of MLP is unlikely to contribute to gene expression changes upon nerve injury, but the protein might rather be involved in structural modification of the cytoskeleton.

### Predominant MLP expression in nociceptive DRG neurons

**Figure 3:**
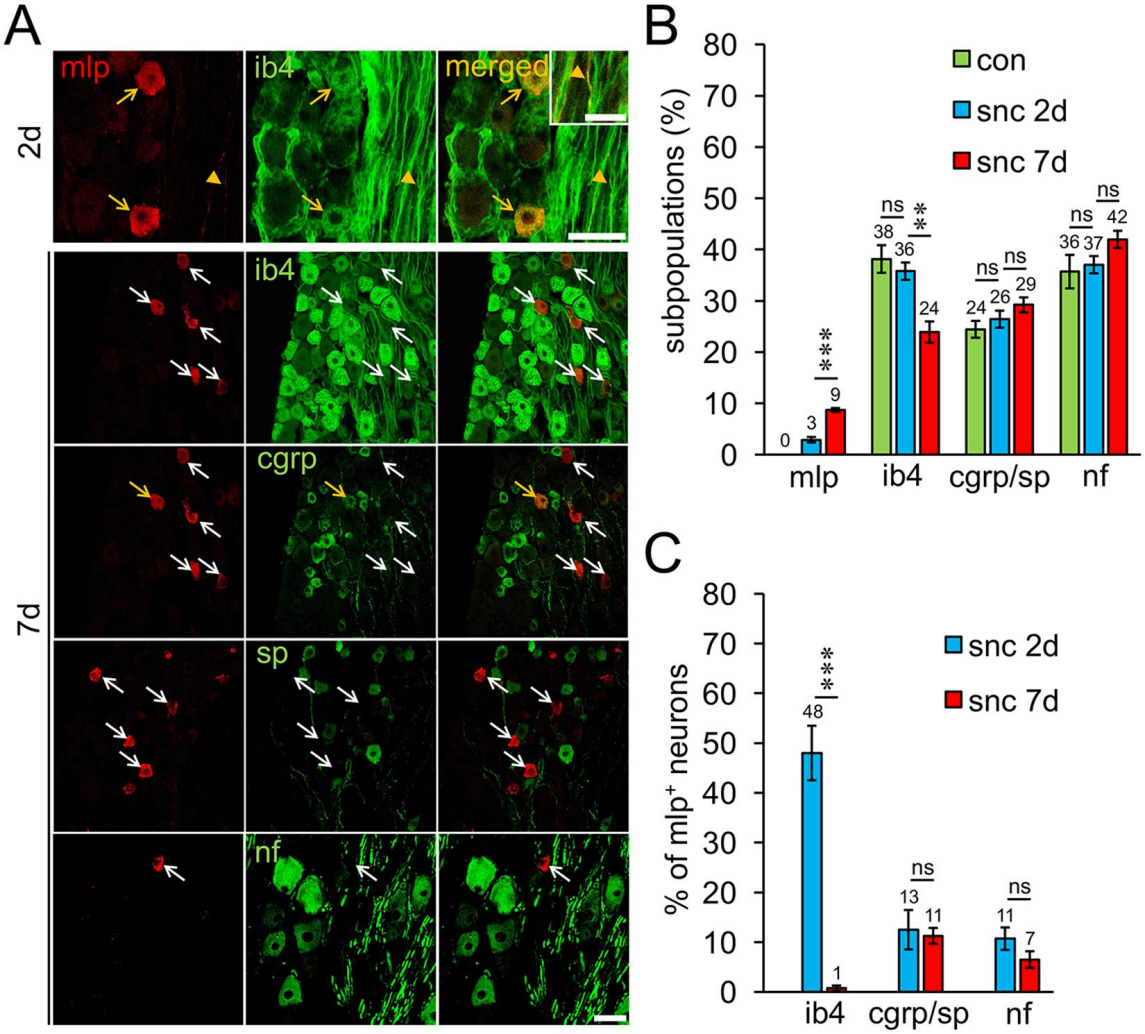
MLP is mainly expressed in nociceptive DRG neurons. (**A**) L4/L5 DRG sections prepared 2 and 7 days after sciatic nerve crush (snc) were co-stained for MLP (red) and one of four markers of neuronal DRG subpopulations (green): isolectin B4 (ib4), calcitonin gene related peptide (cgrp), substance P (sp) or 200 kDa neurofilament (nf). Many MLP-positive DRG neurons (yellow arrows) and their axons (yellow arrowhead) were positively stained for IB4 at 2 days after snc. In contrast, only very few double-positive cells (yellow arrow) were found at 7 days after snc, while most MLP-positive neurons did not co-express any of the four canonical markers (white arrows). Co-staining of MLP for IB4 and CGRP 7 days after snc was performed as triple staining on the same DRG section. Original blue color of CGRP signal was changed to green for a better visualization of positive cells. Scale bars: 50 µm, 25 µm for insert showing higher magnification of a MLP/IB4 double-positive axon. (**B**) Quantification of mlp-, ib4-, cgrp/sp- and nf-subpopulations in DRG from either untreated adult male rats (con) or at 2 and 7 d.p.c. in relation to all βIII-tubulin-positive neurons. Data represent mean percentages ± SEM from 2 rats per experimental group and 4 sections per DRG. Only the subpopulations of MLP- and IB4-positive neurons changed after sciatic nerve crush. (**C**) Percentage of neurons double-positive for MLP and either ib4, cgrp/sp or nf on sections as described in (**A**) at 2 and 7 days after snc. Treatment effects: **p < 0.01, ***p < 0.001, ns: non-significant.

We next investigated whether MLP might be exclusively expressed in one of the three major subpopulations of DRG neurons. To this end, DRG sections prepared 2 or 7 days after SNC were co-stained for MLP and either IB4 (marker for non-peptidergic nociceptors), CGRP+SP (markers for peptidergic nociceptors) or 200 kDa neurofilament (marker for mostly large-sized neurons with myelinated fibers). These canonical markers labeled most neurons in L4/L5 DRG from uninjured rats: 38% of all βIII-tubulin-positive neurons were co-stained for IB4, 24% for CGRP or SP and 36% for neurofilament (Fig. 3A,B). With respect to MLP-positive neurons, only few (13% and 11%) were co-stained for CGRP/SP and neurofilament, respectively, at 2 days after SNC and this proportions were not significantly changed at 7 d.p.c. (Fig. 3C). Surprisingly, while 48% of MLP-positive neurons and their axons were co-stained for IB4 at 2 d.p.c. (Fig. 3A), this percentage strongly decreased to 1% at 7 d.p.c. (Fig. 3C). Thus, the vast majority (81%) of MLP-positive neurons did not co-localize with any of the applied markers 7 days after SNC (Fig. 3A,C). As the number of MLP-expressing neurons is still increasing from 2 to 7 d.p.c. (Fig. 1C), we speculated that DRG neurons might lose their canonical marker label. Indeed, the percentage of IB4-positive neurons significantly decreased 7 days after SNC (24%) compared to 2 d.p.c. (36%) and uninjured controls (38%), while the proportion of CGRP/SP- and neurofilament-positive DRG neurons was unchanged (Fig. 3B). In conclusion, MLP expression seems to be predominantly induced in non-peptidergic nociceptors that lose their ability to bind IB4 upon axotomy^34^. Consistently, cell size analysis revealed that most of MLP-positive neurons were of small-medium-sizes (Suppl. Fig. 1B), being in the range previously described for nociceptive neurons^35^. Additionally, we analyzed MLP expression in tyrosine hydroxylase (th)-positive DRG neurons. This is another subpopulation of neurons that are also negative for the classical canonical markers, but only found in L5 DRGs^36, 37^. However, these th-positive neurons did not express MLP, either (Suppl. Fig. 1C).

**Figure 4:**
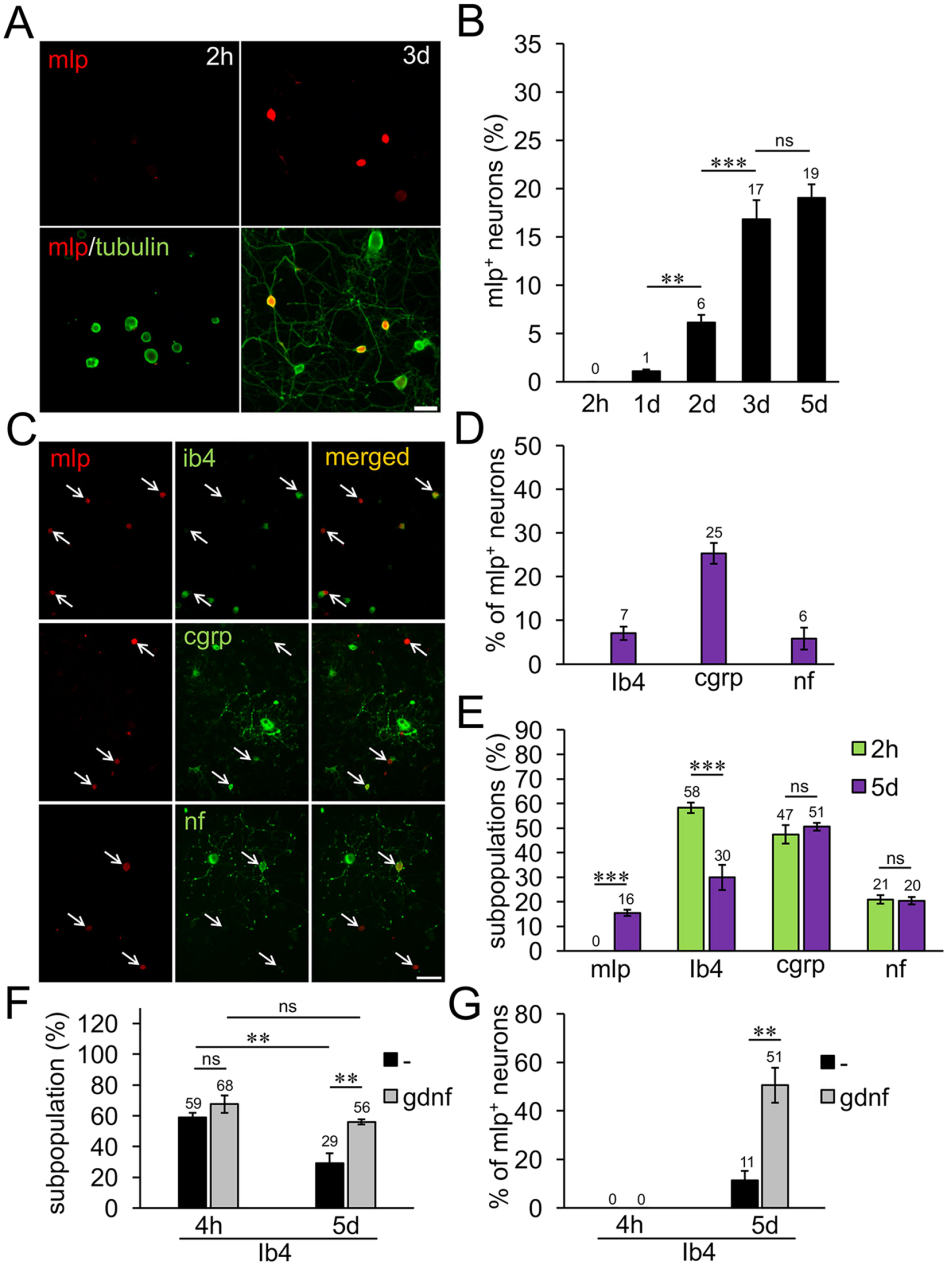
Induction of MLP expression in nociceptive DRG neurons in culture. (**A**) Cultures of dissociated adult rat DRG neurons were stained for MLP (red) and βIII-tubulin (green) 2 hours (2 h) and 3 days (3 d) after cell preparation. Scale bar: 50 µm. (**B**) Quantification of MLP-positive (mlp^+^) DRG neurons in cultures as in (**A**) at 2 h and 1–5 days after plating. Data represent mean percentages ± SEM of 2 independent experiments each with 3–4 repeat wells. (**C**) Representative pictures of cultures co-stained for MLP (red, arrows) and either calcitonin gene-related peptide (cgrp), isolectin B4 (ib4) and 200 kDa neurofilament (nf) (green) after several days in culture. Scale bar: 100 µm. (**D**) Percentage of neurons double-positive for MLP and either ib4, cgrp or nf in DRG cultures as described in (**C**) at 5 d after plating. (**E**) Quantification of mlp-, ib4-, cgrp- and nf-subpopulations in relation to all βIII-tubulin-positive neurons as described in (**C**) at 2 h and 5 d. (**F**) Quantification of IB4-positive neurons cultured either with or without glial-derived neurotrophic factor (gdnf) for 4 h and 5 d after plating. (**G**) Quantification of MLP-positive neurons co-stained with IB4 in cultures as described in (**F**). Treatment effects: **p < 0.01, ***p < 0.001, ns: non-significant.

To substantiate these findings, we also investigated the induction of MLP expression in mixed cultures of cervical, thoracic, lumbar and sacral DRG of unlesioned controls. Consistent with the *in vivo* data, no MLP expression was detected 2 hours after culture preparation (Fig. 4A,B). First positive cells (~1%) were detected at 1 day in culture and their number increased to ~18% at 3–5 days in culture. Of these MLP-positive neurons, 25% were co-stained for CGRP, 6% for neurofilament and 7% for IB4, leaving 62% unassigned after 5 days in culture (Fig. 4C,D). Again, the percentage of IB4-positive neurons markedly decreased with prolonged culturing time (30% at 5 days in culture compared to 58% at 2 h after plating), while the proportion of CGRP- and neurofilament-positive cells remained unchanged (Fig. 4E). Thus, MLP expression was also induced mainly in formerly IB4-positive neurons. In order to substantiate this hypothesis, we exposed dissociated DRG cultures to glial-derived neurotrophic factor (GDNF) as this treatment reportedly delays axotomy-induced downregulation of IB4 detection^34^. Consistently, GDNF treatment significantly increased the percentage of IB4-positive neurons at 5 days in culture compared to untreated controls (Fig. 4F). At the same time the percentage of MLP-positive neurons co-labeled for IB4 increased from 11% to 51% (Fig. 4G), indicating that the vast majority of MLP-expressing neurons gradually lost their ability to bind IB4. The total number of DRG neurons was not affected by GDNF-treatment (Suppl. Fig. 1D). Altogether, these data suggest that MLP was predominantly induced in non-peptidergic nociceptors upon axotomy and might serve as a new marker for this subpopulation upon decline of IB4-staining.

## Discussion

The current study shows for the first time that MLP expression in mature neurons upon axotomy. However, only a small percentage of DRG neurons expressed this protein, most of which represented non-peptidergic nociceptors. As this neuronal subpopulation lost its IB4 staining over time after injury, MLP could serve as a new marker to track the fate of these axotomized neurons.

Previous studies already reported upregulation of Mlp-RNA in adult rat DRG neurons upon partial sciatic nerve ligation^1^ or after sciatic nerve crush^2^ based on *in situ*-hybridization analysis. MLP induction was estimated in roughly 30% of L5 DRG neurons^1^, but no data on protein levels or specific expression in neuronal subtypes were provided. To address the issue whether MLP transcripts are at all translated in neurons, the current study investigated the temporal and spatial expression pattern of the protein in DRG neurons after complete sciatic nerve crush using specific MLP antibodies^3^. Immunohistochemical analysis confirmed gradual induction of MLP protein in maximal 10% of L4/L5 DRG neurons within the first 4 days after injury. Thus, its expression is obviously restricted to a relatively small percentage of neurons *in vivo*, only a relatively small. In cell culture, slightly more (17%) neurons were positive for MLP, which might be due to the cultivation of additional (cervical, thoracic and sacral) DRG neurons compared to the analysis of L4/L5 DRG in *vivo* or the specific culture conditions. However, quantifications always remained significantly lower than 30% and was mainly restricted to small-diameter, nociceptive neurons. In contrast, Newton *et al*. reported MLP expression in DRG neurons of all sizes. This discrepancy might be based on different experimental conditions of the two studies. While Newton *et al*. used a partial ligation model, a different rat strain and *in situ*-hybridization, we performed complete sciatic nerve crush, used Wistar rats and analyzed the protein rather than mRNA expression. Nevertheless, we can neither exclude the possibility that under different experimental conditions MLP might be expressed in other neuronal cell types nor that MLP expression might be posttranslationally regulated. Albeit these remaining open questions, the data presented in the current study demonstrate that significant levels of MLP protein are expressed only in a small and distinct subpopulation of axonally injured DRG neurons.

We initially found that the vast majority of MLP-positive DRG neurons could not be assigned to any of the well characterized canonical DRG subpopulations at 7 days after SNC. However, sections that were prepared 2 days after SNC, when MLP expression had not yet reached maximal levels, approximately 50% of MLP-positive neurons co-labeled by IB4. It is well known that non-peptidergic nociceptive neurons gradually lose their ability to bind this lectin upon axotomy, making their identification difficult. Hence, most MLP-positive neurons detected 7 days after sciatic nerve injury likely originated from these nociceptive neurons. Consistently, the vast majority of MLP-positive neurons in culture could also not be detected by any of the canonical subpopulation markers 5 days after plating. Correspondingly, the proportion of IB4-positive neurons was dramatically reduced at this time point compared to 2 hours, while CGRP and 200 kDa neurofilament expressing subpopulations remained unchanged. To substantiate the hypothesis that most MLP-positive neurons originated from IB4-positive cells we treated the cultures with GDNF. Consistent with Bennett *et al*., GDNF treatment strongly delayed the decline of IB4-staining in MLP-expressing neurons, thereby confirming that the MLP-positive cells express a functional GDNF receptor, which is another typical phenotypic feature of non-peptidergic DRG neurons^34, 38, 39^. Thus, the vast majority of MLP-positive neurons are non-peptidergic, nociceptive neurons.

An important question that needs to be addressed in the future is the functional role of MLP in these neurons. In myocytes, MLP has been shown to interact with at least 16 different partners thereby modulating various cellular functions^7, 10, 40^. In particular, the nuclear accumulation of MLP has been shown as a response to mechanic stress^6, 14^ and described being involved in processes regulating gene expression^5^. However, a similar role appears unlikely in neurons based on our finding that MLP protein was not detected in neuronal nuclei. Nevertheless, MLP could affect other injury-associated processes in DRG neurons, such as axon regeneration. In fact this possibility was already previously addressed using a knock-down approach with Mlp-RNA in cultured DRG neurons with no effect on axon growth^2^. However, based on our findings that MLP is only induced in so few neurons, we consider the detection of a potential effect in these studies impossible as a significant effect would be masked by the majority of negative neurons. A more detailed analysis considering this aspect may therefore lead to a different outcome. As MLP is predominantly expressed in nociceptors, an involvement in the transmission of noxious stimuli, which is altered in injured DRG neurons, is also conceivable^41^. These questions still await further analysis in the future. Nevertheless, our findings identify MLP as a novel marker for a subset of axotomized nociceptive rat DRG neurons that are not clearly recognizable by other established subpopulation markers.

## Electronic supplementary material

Supplementary Figure 1
