# Supplementary material for: Nociceptive DRG neurons express muscle lim protein upon axonal injury
Source: Sci Rep. 2017 Apr 4;7:643. doi: 10.1038/s41598-017-00590-1 (PMC5428558; doi:10.1038/s41598-017-00590-1)

Nociceptive DRG neurons express muscle lim protein upon axonal injury

Evgeny Levin, Anastasia Andreadaki, Philipp Gobrecht, Frank Bosse and Dietmar Fischer

Supplementary Figure 1

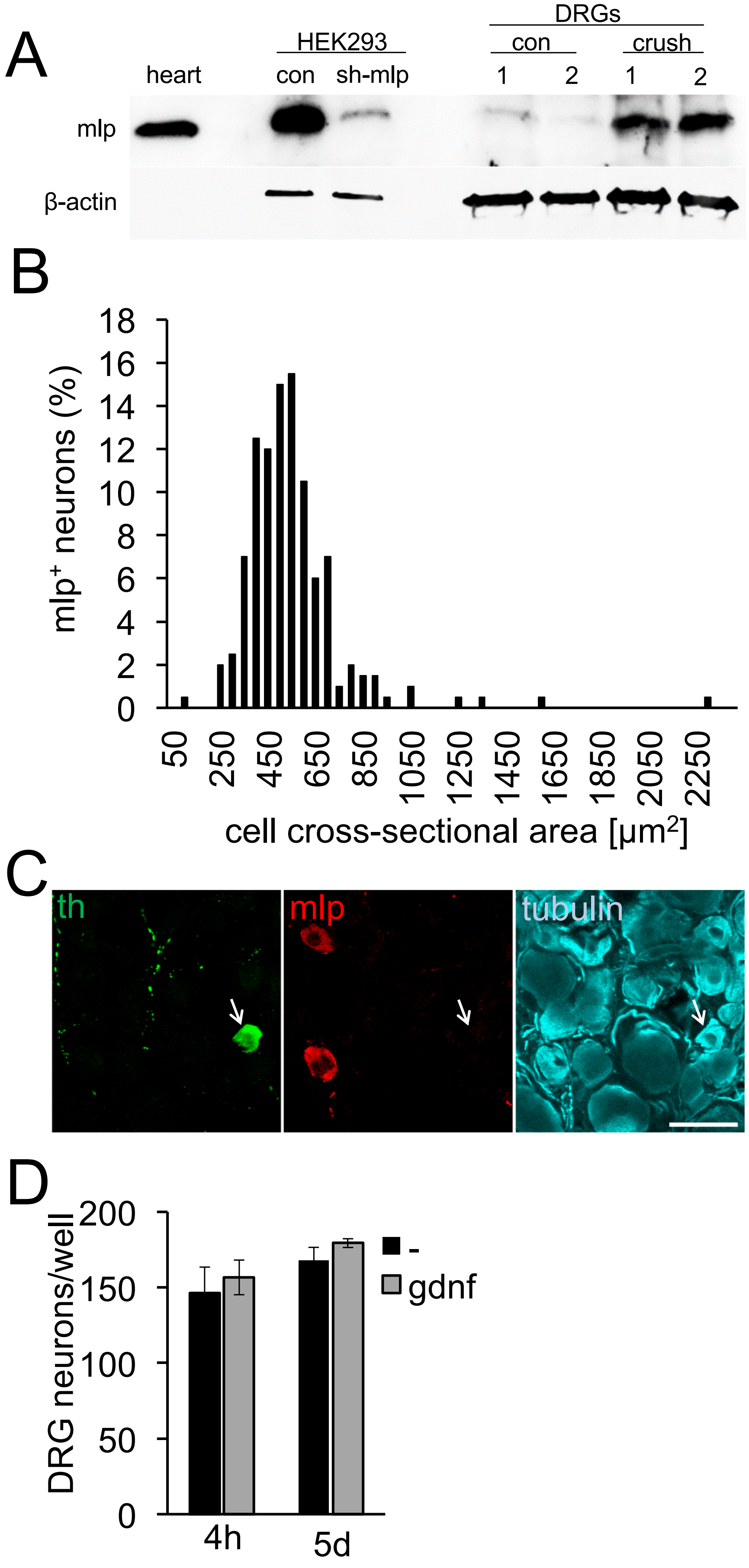

Supplement: Supplementary file 1 — Supplementary Figure 1 [file 41598_2017_590_MOESM1_ESM.pdf]
